# 4523 Assessing Depression in Puerto Rican Hispanic Patients Hospitalized with Heart Failure

**DOI:** 10.1017/cts.2020.256

**Published:** 2020-07-29

**Authors:** Ariel Gonzalez-Cordero

**Affiliations:** 1 University of Puerto Rico-Medical Sciences Campus

## Abstract

OBJECTIVES/GOALS: Heart failure and depression are important public health problems. Depression has been identified as an independent risk factor for increased cardiovascular morbidities. It is estimated that 1 out of every 5 patients living with heart failure suffer from depression. (Kop, W. J.) Studies have found that Hispanic patients have a higher rate of heart failure than non-Hispanic whites. Current guidelines recommend that proper screening tools must be used to identify and manage major depression disorder in HF patients, however many of these patients go unrecognized in the medical setting. The prevalence, management, and outcomes of depression among PuertoRican Hispanics living with heart failure is unknown. Objective: The purpose of this study is to evaluate the relationship between heart failure and depression in Hispanics with heart failure METHODS/STUDY POPULATION: To this end, we will perform a secondary analysis of data from the PR CardiovascularSurveillance Study (PRCSS). We will extract personal data from 4,461 medical records of patients admitted with heart failure (ICD-9 Codes 428) at 21 hospitals in Puerto Rico, during the years 2007, 2009 and 2011. For statistical methods, we will implement chi-square and t-tests at a significance level of 0.05 RESULTS/ANTICIPATED RESULTS: We expect to see older aged women with higher NYHA and pro-BNP levels to be associatedwith diagnosis of major depression disorder and worse in-hospital outcomes DISCUSSION/SIGNIFICANCE OF IMPACT: With this study, we would like to raise awareness about depression in patients with heart failure, and its role in improving patient outcomes. Moreover, we would like to determine if there are gender-specific health disparities among Puerto Rican Hispanics with heart failure

